# Alcohol Consumption and Perceptions of Health Risks During COVID-19: A Qualitative Study of Middle-Aged Women in South Australia

**DOI:** 10.3389/fpubh.2021.616870

**Published:** 2021-04-26

**Authors:** Belinda Lunnay, Kristen Foley, Samantha B. Meyer, Megan Warin, Carlene Wilson, Ian Olver, Emma R. Miller, Jessica Thomas, Paul R. Ward

**Affiliations:** ^1^College of Medicine and Public Health, Flinders University, Adelaide, SA, Australia; ^2^School of Public Health and Health Systems, University of Waterloo, Waterloo, ON, Canada; ^3^School of Social Sciences, University of Adelaide, Adelaide, SA, Australia; ^4^Fay Gale Centre for Research on Gender, University of Adelaide, Adelaide, SA, Australia; ^5^School of Psychology, University of Adelaide, Adelaide, SA, Australia

**Keywords:** alcohol, women, middle-aged, pandemic, risk, breast cancer

## Abstract

Australian women's alcohol consumption has increased in frequency during COVID-19. Research suggests this is to cope with stress resulting from the pandemic and COVID-19 countermeasures that require social distancing. This is a critical public health concern because increased alcohol consumption, even for a short period, increases the myriad longer-term health risks associated with cumulative exposure to alcohol. This paper provides unique qualitative evidence of how health risk perceptions are re-focused toward the shorter-term during the pandemic, through analysis of interviews with 40 middle-aged Australian women (aged 45–64) representing a range of self-perceived drinking status' (“occasional”/“light”/“moderate”/“heavy”) before and then during the pandemic (*n* = 80 interviews). Our analysis captures women's risk horizons drifting away from the uncertain longer-term during COVID-19, toward the immediate need to “get through” the pandemic. We show how COVID-19 has increased the perceived value of consuming alcohol among women, particularly when weighed up against the social and emotional “costs” of reducing consumption. Our findings have implications for the delivery of alcohol-related health risk messages designed for middle-aged women both during, and into the recovery phases of the pandemic, who already consume more alcohol and experience more alcohol-related health risk than women in other age groups.

## Introduction

Women in midlife (aged between 45 and 64 years) typically consume alcohol more than any other age group (1) and alcohol intake, in addition to age, is associated with increased risk for various long-term health issues (1). Consequently, a critical public health concern follows from recent data demonstrating that Australian women have increased their frequency of alcohol consumption (number of days since the last drink) since the acknowledged emergence of COVID-19 in Australia in March 2020 (2). Discussion surrounding the impacts of COVID-19 on health, and social issues more broadly, would suggest that the increased frequency of alcohol consumption is a gendered issue, with women feeling the effects of the pandemic in different and more pronounced ways relative to men (3). National survey data show that women report different reasons for increases in alcohol consumption than men (2). In their study of the impacts of COVID-19, Biddle et al. (4) found a negative relationship between mental health and alcohol generally, and relative to men, Australian women have experienced greater mental health instability during COVID-19 (5). Feeling stress is the most common reason women provided for their increased alcohol consumption during COVID-19 (2). Of compounding concern is survey data that shows loneliness is the most common personal stressor experienced during COVID-19, with Australian women more likely than men to report feeling lonely (4, 6). This is worrisome given our previous research which showed women typically consume alcohol to cope with loneliness and unhappiness, and to reduce stress (7, 8).

Myriad compounding factors have increased mental health instability for women during lockdown conditions. Due to the pandemic, women have spent more time at home and this is associated with numerous and significant gendered stressors. Relative to men, women experienced greater increases in caring responsibilities and unpaid work during COVID-19 (5, 9). For women aged 45–64 years (our study population) this translated to working from home alongside “juggling” support for older children in the later stages of school or enrolled in university and care for elderly relatives. While “stay at home” messages are clearly important during the pandemic, home cannot be taken-for-granted as a “safe haven.” Home is not always a calm or safe space for women, as is represented in the increased incidence of domestic violence (10) and increased self-reported experience of isolation (4). The unequal gendered impacts on women resulting from COVID-19 also extends to their participation in the paid labour force. More women have lost their jobs during COVID-19 than have men, reflecting the disproportionate representation of women employed casually and in industries adversely affected by COVID-19 countermeasures (e.g., hospitality and tourism), resulting in a “pink-collar” recession. Furthermore, the largest fall in hours of paid work was experienced by women because, more so than men, they are employed on a casual basis and in industries where most job losses occurred due to social distancing measures (hospitality, education, and tourism). Additionally, more women than men comprise the Australian health and caregiver workforce (11) and, as such, face disproportionate stress associated with working on the “frontline” during the crisis.

As the pandemic unfolded in Australia, it became evident that measures to abate the spread of COVID-19 entailed unprecedented change in the practise of everyday living. In consideration of the unique gendered effects on women, it is anticipated that COVID-19 countermeasures have and will continue to augment the perceived value of consuming alcohol in women's lives. This paper presents timely insights into how middle-aged women (45–64 years of age) in South Australia describe their perceptions of alcohol-related health risks (and health risks more generally) during the COVID-19 pandemic compared to before the pandemic. The original focus of our research (before COVID-19), was to understand the role of alcohol in women's lives and the extent to which breast cancer risk is factored into consumption. Alcohol is a class 1 carcinogen, which means that the ability for alcohol to cause cancer is certain, and it is an important modifiable cause of breast cancer (8, 12–17). The timing of the pandemic relative to initial interview data collection provided the opportunity to follow-up women previously interviewed about their consumption of alcohol and reasons for consuming alcohol during pandemic “lockdown.”

This type of enquiry is indispensable because we know that the calculability of risk is compromised when the future is incalculable; and in turn negatively affects the relevance of future-oriented public health messaging (18). In so far as alcohol is concerned, we know that the uptake of alcohol risk-based messaging (e.g., reduce and consume at recommended levels) hinges on women's evaluation of scientific evidence weighed up against their “lay” health knowledge and experiences (19–21). This knowledge creates a theory of disease causality that has influence on women's reasons for continuing or modifying alcohol consumption (8). Our insights on the effect of the pandemic on women's alcohol consumption and health risk perceptions are crucial for future risk messaging both during COVID-19 and into the recovery phases of the pandemic.

## Method

A study exploring women's lay knowledge of alcohol and breast cancer risk was undertaken between July and December 2019. This involved interviews with 51 English speaking middle-aged (45–64 years) South Australian women of mainly Anglo-Saxon ethnicity (by researchers KF and BL) who identified reasons for alcohol consumption and understanding of breast cancer risk before COVID-19 emerged in Australia. Women were purposively sampled to vary in alcohol consumption by “self-perceived drinking status” to capture “occasional,” “light,” “moderate,” and “heavy” drinkers across a cross-section of ages (comprising the 45–64 demographic bracket) income levels and education. Self-perceived alcohol consumption was used given our study focus being to understand the functions and meanings of alcohol consumption relative to women's own perceived breast cancer risk. Our sampling strategy involved purposeful selection of mostly self-perceived “light” and “moderate drinkers,” and “extreme” case sampling (22) of some “occasional” and “heavy” drinkers for variation (see Table 1). Sampling continued until saturation was reached (23, 24). During COVID-19 (March to April 2020) follow-up interviews were conducted with 40 of the initial 51 participants, with the aim to explore the impact of the significant health risk and life challenges faced in the pandemic on their alcohol consumption and risk perceptions. For the purposes of this paper, we are not examining any differences in women's responses by demographic grouping, as our focus is on drinking status in so far as to achieve an understanding of if and/or how the pandemic has impacted alcohol-related health risk perceptions.

**Table 1:** Sample characteristics by self-perceived alcohol consumption.

**Self-perceived drinking status**	**Interviewed**
Occasional drinker	5
Light drinker	15
Moderate drinker	15
Heavy drinker	5

*Self-perceived drinking status was identified through responses to a participant survey conducted before COVID-19*.

Before COVID-19, interviews explored women's perceived associations between alcohol and breast cancer risk specifically. Women's “lay expertise” around rationalities for consuming alcohol were explored, particularly as they related to evaluation of longer-term health risks, using breast cancer as the example of an outcome to be avoided. We also explored participant–driven explanations for alcohol consumption, and justifications of the evidence used for the belief (or not) that alcohol is a modifiable risk factor for breast cancer, including trust in different sources of evidence and risk messaging. Finally, barriers and enablers to intake modification were examined and their relationship to potential risks. At follow-up interviews during COVID-19 participants were asked if social distancing rules changed how they socialised or connected with others, and in what ways. Social distancing rules to prevent the spread of COVID-19 resulted in restrictions on public activities and were implemented by the South Australian Government in March 2020 and were gradually easing by October 2020.^1^ Different states and territories of Australia experienced the pandemic differently and accordingly, Government responses varied by locality. The impacts of social distancing on participant's alcohol consumption, if they had stockpiled alcohol, whether the reasons why they consume alcohol had changed and if any changes in consumption patterns had occurred during COVID-19 were also discussed. Finally, participants were asked whether their perceptions of risk had changed and about possible shifts in their balancing of long term vs. short terms harms and gains. The complete interview schedule can be requested from the corresponding author.

The two waves of interviews (before and during COVID-19) were conducted slightly differently due to the social distancing conditions experienced during COVID-19. Interviews conducted before COVID-19 were ~1 h long and in person by the researchers KF and BL (both female and experienced in interviewing) in participant's homes, cafes or community centres, public libraries, whatever was preferred by the participant. The interview followed a schedule, with the researcher probing for further detail on women's thinking, logic and perspectives regarding alcohol and breast cancer. It was emphasised that there were no incorrect answers to any of the questions asked and this supported rapport-building during the interview. The researcher approached the interviews with “empathic neutrality” (22) and worked hard to avoid moralising any information women contributed. Participants were not sent the interview schedule prior to the interview, because in this wave of interviews we wanted to explore women's lay knowledge about alcohol and breast cancer. We hypothesised that sending them the schedule might encourage them to view the interview more formally and research the link between alcohol and breast cancer prior to the interview. The follow-up interviews conducted during COVID-19 lasted 30–45 min and were undertaken by researcher BL over the telephone or via tele/videoconference applications (as per social distancing rules) following a semi-structured schedule. Participants were emailed to establish an interview time and preferred mode of communication. In these follow-up interviews, women were sent the schedule prior to the interview (1–2 days ahead) to allow time for reflection before they provided a response, and as not to cause undue pressure during the crisis.

All interviews were audio-recorded, transcribed, and then wave 1 and wave 2 transcriptions from each participant were matched and de-identified (pseudonyms are used in this paper). Once matched, transcripts were analysed cross-sectionally at both time points and then across time, providing the capacity to identify any changes in participant's alcohol-related risk perceptions across time and how these might be linked to the experience of COVID-19. This approach also allowed exploration of the impact of policies and regulations implemented to stem the spread of COVID-19 (i.e., pandemic countermeasures) (25).

Data were managed using QSR NVivo version 12 qualitative data analysis software (26). Cross-sectional analysis followed a three-step progressive method of (1) pre-coding, (2) conceptual and thematic categorisation and (3) theoretical categorisation (27). To check for agreement in coding and improve explanatory rigor the researchers BL, KF, MW, JT and PW each co-coded four transcript pairs comprising interviews conducted before COVID-19 (wave 1) and during COVID-19 (wave 2) per participant; one pair from each category of self-perceived drinking status: “occasional,” “light,” “moderate,” and “heavy.” Once agreement was reached on codes comprising the coding framework – a process that achieved interpretative validity - transcripts were deductively coded against the agreed coding framework and new codes were added as they emerged through analysis following a framework analysis approach (28). Data were then organised by theme into time-ordered, sequential matrices to facilitate comparison of women's reasons and logic for alcohol consumption before COVID-19 with their descriptions of behaviour during COVID-19 and in responses to public health messaging. Analysis focused on how the thematically grouped data in each set of paired transcripts changed or remained stable over time (29) and on the conditions, causes and consequences of change (30, 31). This allowed us to detect patterns of change or where previous risk perspectives were substantiated in participant's interview responses across time, within themes such as precursors of alcohol consumption and participant's experiences; risk perceptions and shifts away from longer-term health risks toward shorter-term outcomes or where the pandemic solidified or confirmed a participant's pre-existing short-term focus.

Both waves of interviews and associated protocols had full ethical approval from the Social Behavioural and Human Research Ethics Committee at Flinders University, South Australia.

## Results

Our results explicate women's perceptions of alcohol-related health risks before and during COVID-19 and show the pandemic shortened most women's risk horizon toward the more predictable short-term. The perceived need to “get through the pandemic” impacted on intended alcohol consumption, particularly when weighed up against the social and emotional “costs” of reducing consumption. Before we present comparisons, it is necessary to first summarise women's risk perceptions described when we interviewed participants before COVID-19. Importantly, for many women it seemed possible to contemplate reducing or even begin to make reductions in their alcohol consumption. We then integrate data from interviews undertaken during COVID-19. In several cases we compare these to responses before COVID-19, to demonstrate how perceptions of risk were re-focused to the more predictable short-term amidst the pandemic and negated previous possibilities for alcohol reduction. Re-interviewing women provided data that described how changed life circumstances impacted women's rationalizations of alcohol consumption, particularly as they related to evaluation of known health risks. For participants of our study, such changes were work-related - generally this included a reduced sense of work satisfaction (through isolation), increased pressure at work and in some cases, a sense of job insecurity (at the time of interviews no participant had been stood down from work). Various women's caring role of older parents increased, although some experienced restricted access to elderly or unwell parents. Several participants needed to increase the care they provided their children with additional needs due to support service shut-downs. Retired participants who typically looked after (and enjoyed looking after) their grandchildren became isolated from them. Such participants also experienced reduced volunteering opportunities and subsequently, a reduced sense of purpose. Many women talked about feeling increased pressure to source and provide food for others in the home (an extension of their care role) and having sole responsibility for maintaining the domestic space. While women in our sample who had children mostly parented older children, they spoke about needing to manage disruptions to their children's vocations and some talked about their children either returning home to live or being isolated because they were staying with a partner and were unable to move between houses due to distancing rules. Many women spoke about gym closures and reductions in personal training/boot camps which adversely impacted their sense of connectedness with the community and their health/well-being, and their weight. As can be seen by this extensive albeit not exhaustive overview, the magnitude and implications of change obviously varied across the sample, yet no participant's life circumstances were unaffected by COVID-19. In our results we also include data to show how “other” risks emerged alongside the pandemic that took precedence over any considerations of longer-term alcohol-related risk. For some women, the pandemic provided “evidence” that substantiated their pre-existing short-term or even fatalistic philosophy (i.e., “life is short” “anything can happen” and accordingly we should “live in and for the moment”). Several women were already leading limited lives economically and socially that necessitated a short-term focus before COVID-19 emerged and the pandemic ensued little change. The final section of our results shows how women questioned the sustainability of change required for longer-term health risk reduction and in the context of “other” risks perceived as more urgent.

### Perceptions of Alcohol-Related Health Risk Before the Pandemic

Interview data collected before COVID-19 indicated that critical distinctions were made between women about the link between alcohol consumption and breast cancer risk. Such distinctions included differentiated reasons and rationalities for alcohol consumption that linked to women's “lay knowledge” of what causes breast cancer and their perceptions of their own breast cancer risk. Interviews also indicated two prevailing decision dilemmas. The first focused on women deciding on whether they were *willing* to reduce consumption and the second involved their assessment of the *feasibility* of achieving this goal. Most participants could make sense of scientific information about the negative impact of alcohol on health and, before COVID-19, acknowledged it as a risk factor for breast cancer. Notwithstanding this, women rationalised consumption according to a range of social, cultural, financial and emotional considerations (8). Some women expressed willingness to consider reducing alcohol consumption and described self-directed measures to reduce or moderate their alcohol intake (such as keeping drinking diaries, creating rules that permitted drinking alcohol only on certain days, and so on). The COVID-19 pandemic, and the associated social distancing and home-based isolation designed to drive down COVID-19 case numbers, created a massive change to the way participants lived their daily lives. These changes impacted women's feelings about the feasibility of reducing alcohol consumption and seemed to justify continued consumption. For participants who contemplated reducing alcohol consumption with the view of reducing breast cancer risk, COVID-19 had a negative effect. Further detail on views of women's logic in navigating alcohol-related breast cancer risk can be found elsewhere (8).

### Shifting Risk Perceptions Toward the Short-Term During COVID-19

In terms of risk-based decisions, the ambiguity experienced during “pandemic life” seemed to curtail participants' longer-term concerns. Positive feelings gained from drinking alcohol justified consumption. That is, relative to the certainty and immediate relevance of adapting and responding to public health guidance and “survive the crisis,” the value of consuming alcohol as a coping mechanism took precedence when held up against the *possibility* of health risks. One participant rationalised consuming alcohol by detailing the positive impact on mood gained from drinking; “*there is no point being alive if you're miserable”* (Rebecca, aged 47, moderate drinker, during COVID-19, partnered, no children). In fact, when asked about longer-term risks, most women saw this as of only minor concern; “*when you take a whole picture of life one glass of wine seems insignificant”* (Tricia, age 59, light-drinker, during COVID-19, divorced, children). The prominence of participant's concerns before COVID-19 about longer-term health risk (using breast cancer as an example) was also reduced by the experience of the pandemic and replaced by the more pressing concerns of vulnerability to the virus. For example, before COVID-19 Joy suggested she would be willing to follow guidelines about levels of alcohol consumption for health risk reduction, using the example of breast cancer risk reduction, she said:

“*If you're going to tell me that I'm going to get breast cancer from the next rum I have, then I'm probably not going to have the next rum” (Joy, aged 50, moderate drinker, divorced, one child, before COVID-19)*.

In contrast, during the pandemic, Joy's risk perspective was re-focused away from the longer-term risks (i.e., breast cancer risk) toward the “immediate concern” of getting through the pandemic. This was pronounced for her due to pre-existing health conditions that would be co-morbidities if she contracted COVID-19. For example:

“*My immediate concern is obviously what's going on because I'm in that high-risk group [for COVID-19]. So, things like breast cancer and things like that they've sort of been put on the back burner, if that makes any sense. I'm sort of at this point where I'll get through this and then I'll worry about that [alcohol consumption]” (Joy, aged 50, moderate drinker, divorced, one child, during COVID-19)*.

Other participants did not discount their knowledge that alcohol consumption impacts longer-term health risk, using the example of breast cancer risk. Nonetheless, the time horizon of their health decisions was adjusted in response to the crisis, to focus on the current pandemic demands and threats. During COVID-19 participants were exposed to a new and pressing health risk; a contagious and life-threatening virus and this became an equivalent or more dominant focus. For example, Stephanie contemplated reducing consumption with the view of protecting herself from illness before COVID-19. When asked before COVID-19 how she thought she could reduce her risk of illness (specifically breast cancer), she responded:

“*probably cutting down on drinking alcohol, well, I already have but I had been thinking about giving it up completely” (Stephanie age 48, light drinker, divorced, no children, before COVID-19)*.

During COVID-19 however her previous consideration given to reducing alcohol consumption was expanded to also include immediate concerns and ways to prevent contracting SARS-Cov-2:

“*I haven't thought any more about breast cancer. I did think about alcohol and the relationship between alcohol and a whole range of things; breast cancer's one of them but then other cancers, colon cancer, whatever, and even kidney stones; you're meant to be drinking lots of water and alcohol is not good for it. But I'm certainly thinking about the risk of COVID-19 and where I go and I've got my hand sanitiser and I'm washing my hands and things like that” (Stephanie, age 48, light drinker, divorced, no children, during COVID-19)*.

Several participants described reducing consumption during COVID-19 – but this was not motivated by health risk reduction, but rather by maintaining or bolstering their ability to cope during the pandemic. For example, Alex, who through COVID-19 worked from home and felt shut off from her colleagues explained:

“*I didn't see that as a response that I would drink more but I thought it was better if I was actually drinking less, I'd be healthier and less anxious and it would be better for my mental health if I really restricted alcohol, so, I didn't see that I would be drinking more wine, I just thought I need to drink less during this time” (Alex, aged 64, moderate drinker, partnered, children, during COVID-19)*.

In this instance, the acute nature of viral risk resulted in a change in alcohol consumption, whereas before COVID-19 when asked about if breast cancer risk and reducing alcohol consumption was on her radar Alex's response showed reduction as a possibility, though notably this was not actioned:

“*it certainly was prominent in my thinking about if I'm drinking in a risky way I need to cut that back. Like I've seen people go through chemo and radiation therapy and it's not something I fancy so if I can not be smoking as a risk factor for lung cancer I can cut back my drinking to a degree as a reduction of risk factor for getting breast cancer, for instance.” (Alex, aged 64, moderate drinker, partnered, children, before COVID-19)*.

Other participants spoke about *intentions* to limit alcohol consumption to improve their physical health, but the rationale was physical fitness to withstand the COVID-19 virus rather than for breast cancer considerations. For example:

“*There's a link between wanting to be fit so that if I got the virus I start from a good place and then kind of drinking that in and going in long term I want to be fit and this –obviously there are things in the world that can kill me that I have no control over and some of those are cancer” (Anna, aged 46, moderate drinker, married, children, during COVID-19)*.

For some participants, the notion of risk as a broad consideration, not necessarily focused on health, intensified during COVID-19 and this seemed to manifest in more frequent alcohol consumption. For example, women described risks to maintaining work outputs despite pandemic impacts. For example, before COVID-19 Nadia, a self-perceived light drinker explained:

“*The one thing I like to do each day when I get home is separate that work from my time so I'll have one beer. Generally I try and have two alcohol free days a week, completely, but I'll, probably three or four nights of the week, have just that one beer and that's it; that's all I have and don't touch anything else” (Nadia, aged 49, light drinker, separated, children, before COVID-19)*.

During COVID-19, Nadia's alcohol-free days disappeared:

“*So, I guess I'm kind of playing that off and rewarding myself, and saying, well, you can have a drink each day….so definitely more consumption, but obviously less socializing and going out doing that.” (Nadia, aged 49, light drinker, separated, children, during COVID-19)*.

During COVID-19, participants' responses to probes about alcohol-related risks highlighted that hard times make it difficult to focus on longer-term possibilities. During COVID-19, women provided explanations like the following:

“*[people are] focused on solving the immediate issue” (Donna, aged 60, occasional drinker, living alone, no children, during COVID-19)*.

Another example is:

“*I think it's just really focused in on the here and now, rather than the longer-term of anything, really” (Paula, aged 48, light drinker, married, children, during COVID-19)*.

It was clear from several participants' accounts of the all-consuming nature of existence through the pandemic (including processing news, deciphering public health information, coordinating and adapting to new ways of living), that little headspace was left for comprehending longer-term health risks. This shaped participants' orientation toward the immediate future, for example:

“*I don't think I feel shock, but I just feel this is a whole new thing and it is weird, and I think it's quite hard to get your head around. I think that probably slows people down in taking preventative measures that they should be taking” (Lois, aged 60, moderate drinker, married, children, during COVID-19)*.

One participant did discuss her continued awareness of the longer-term impact of her behaviours through COVID-19 but moored this in concerns for reducing the burden on the health system. She said:

“*But when you do talk to people – what's the one thing everybody talks about? They just talk about this – the COVID-19. So it feels like the whole world is in limbo, waiting for something. I think we're just all going through the motions just waiting for it to be over. There probably is no room [to think about breast cancer risk]. And it's like, while this is going on, it's like you don't want to get sick with anything else, because you don't know what the health care system is going through at the moment. Is the health care system overwhelmed? (Tiffany, aged 53, moderate drinker, living alone, no children, during COVID-19)*.

Tiffany's comment about there being “*no room*” to contemplate breast cancer risk again points to the difficulty comprehending longer-term risks in the frame of pandemic health risks:

“*You feel like, well, I don't really want to get sick with anything else, because it's probably going to be too hard to try to get treatment for anything else” (Tiffany, aged 53, moderate drinker, living alone, no children, during COVID-19)*.

Many of the women we interviewed described having no coherent view of the longer-term future during COVID-19, and expressed feeling “*in limbo,”* waiting to see how the virus “*played out*,” including determining how public health responses to COVID-19 could impact their life. This climate of uncertainty not only changed women's feelings about the feasibility of reducing alcohol consumption, but adversely affected their willingness to make modifications. Conversely, during COVID-19 women were able to describe the myriad “good things” they gained from consuming alcohol; relief from stress or boredom, reward for coping, retaining a sense of normality, and facilitating a sense of connection with others through the shared activity of drinking alcohol or through using alcohol consumption as a talking point.

### The Emergence of Other Risks During the Pandemic: Reduced Attention on Alcohol-Related Risks

Many participants indicated that concerns about other people's health risk took precedence over worry about their own health, and concern about managing any longer-term alcohol-related health risks. This concern was primarily for elderly parents and loved ones working in occupations involving close contact with others during the pandemic. The following excerpt captures how priorities around risk were not about personal risk but risks to others, and the risk of not being able to help:

“*I don't think it's made me think any differently about any risks really to myself, it's more if something happened to Mum and she's over there all by herself is probably more a bit of a thing where in the past I've just thought, well, if something happened to mum, well I would jump in the car and drive her which I can still do but if, you know, more lockdown business happens and I couldn't then I haven't quite figured my way through that one…So it's probably more thinking about from her point of view, from my point of view thinking about her and what would happen if it got any worse or if something happened to her rather than myself. I don't think anything would happen to me” (Trudy, aged 60, moderate drinker, widowed, no children, during COVID-19)*.

Additionally, several participants discussed the moral aspects of risk, describing the need to be “seen to be doing the right thing.” For example:

“*to me it's a matter of doing the right thing to be not seen out so that other people don't go out….the risk is not about me getting it [COVID-19], the risk is about doing the right thing in terms of staying away from people” (Gillian, aged 51, light drinker, married, no children, during COVID-19)*.

One participant seemed unable to comprehend risks outside of those imposed by the SARS-CoV-2 virus resulting in the COVID-19 pandemic. This participant interpreted our question on perceptions of alcohol-related health risk during COVID-19 only in the context of the virus, perhaps demonstrating the pervasiveness of viral risk messaging and/or her “blinkered” approach to longer-term health risk. When asked about how COVID-19 had impacted and made her feel about her health she replied:

“*I am conscious about more hand washing, social distancing, disinfecting the trolley once I get that and all little things. It's a bit scary to go to the supermarket these days” (Tamara, aged 45, moderate drinker, divorced, children, during COVID-19)*.

Another participant countered the link between alcohol and longer-term health risks, and rationalised consumption as improving health through COVID-19 by “*easing stress and improving sleep”* (Mary, age 64, moderate drinker, separated, no children, during COVID-19); two factors she believed compromised health substantially and that if improved, would bolster her resistance to the virus and increase her overall longevity.

### Pre-existing Short-Term Focus Substantiated During the Pandemic

Not all women's perception of risk was open to a shift. For some women, the pandemic substantiated their pre-existing preference or need to focus on the present time. Others already lead lives characterised by economic challenges and/or social restrictions, and for them the pandemic provided no change to the previous necessity to focus on “getting through” the difficult short-term. For example, before COVID-19, one participant referred to the “lottery” of health outcomes and rationalised alcohol consumption accordingly. This became consistent both before and during COVID-19, and living through the pandemic confirmed for her that:

“*it's a bit of a lottery really, your health, isn't it? You need to be mindful of doing the right thing, and if you're doing some things like drinking alcohol, it's seen as a bad thing, and you enjoy it, then moderation hopefully it will be okay” (Kimberly, aged 62, moderate drinker, married, children, during COVID-19)*.

Another participant, in a fatalistic sense, suggested that her perceptions of the probability of longer-term ill health have increased because of COVID-19:

“*I think it [COVID-19] probably has heightened your awareness of the fact that you could get other illnesses or disease as well but that's about as far as it goes with me. I think it just highlights the fact that anything can happen health wise” (Lois, aged 60, moderate drinker, married, children, during COVID-19)*.

Where the time horizon that impacted decision-making and alcohol consumption changed after COVID-19; the focus was on meeting immediate needs, be they caring for themselves or others:

*I think it [COVID-19] shows you life is short and just enjoy what you can while you have got your [virus-free] health sort of thing” (Harriette, age 55, heavy drinker, married, children, during COVID-19)*.

This excerpt also reflects a “live for the here and now” philosophy that was echoed by many women interviewed during COVID-19, and for many women interviewed, this involved consuming alcohol at before COVID-19 levels or more frequently.

For some of the women who participated in our study, life circumstances before COVID-19 were already characterised by persistent unease. For women like this, the pandemic had little influence on their alcohol consumption and perceptions of alcohol-related risk. For example, while one participant stated during COVID-19 “*I can tell you my own mortality has surfaced”* (Michelle, age 56, heavy drinker, married, children, during COVID-19) which resulted in “*short-term thinking,”* in terms of her health risk perceptions, nothing changed as a result of COVID-19. There were simply no shifts for her, given before COVID-19 she said with aplomb “*I'm so far down the rabbit hole of risk”* (Michelle, age 56, heavy drinker, married, children, before COVID-19), and she described feeling unable to navigate back out, and that alcohol consumption was a strategy for managing this feeling. Before COVID-19 emerged in Australia, Michelle described feeling “*held hostage by alcohol”* and it seemed that alcohol consumption is so engrained as a way of life, any potential risk is innate and modifying consumption is outside of her control. In this instance, recognizing the physical health risk of consuming alcohol was not enough to motivate reduction.

### Sustainability of Change Required to Reduce Alcohol Consumption and Impacts on Risk Prevention

Before COVID-19, participants spoke about the changes that would be required for them to reduce alcohol-related health risks, and during COVID-19 these became, in participant's words “*too much”* time and accepted as too challenging. The comparatively immediate and less complex routines required to prevent viral transmission (e.g., practising distancing, hand washing) were deemed less problematic as most women envisaged the virus soon disappearing. In fact, commitment to willingness and evaluation of the feasibility of preventing COVID-19 contrasted vividly with the difficulty of reducing breast cancer risk. In the following explanation a participant juxtaposes the complexity of change required to reduce alcohol-related risks with the simplicity of COVID-19 prevention:

“*Because it's just basically, if I self-isolate I'm safe. So, I just have to do one thing, really. Whereas with breast cancer, it's a multi-layered approach to risk…but [for COVID-19] it's really – it's just some things that I just have to think about and it doesn't impact my lifestyle, you know? It doesn't take away from my life. So that's the difference is, I think, is that yes, I can do this for say, three months if I need to, but I couldn't do it for three years. That's just not going to happen” (Rebecca, aged 47, moderate drinker, partnered, no children, during COVID-19)*.

This participant's explanation also captures how consideration is given to the relative difficulty of short vs. longer-term changes. The costs to lifestyle of breast cancer risk management appear to outweigh the investment of time required to practise health prolonging activities. For some participants, the time commitment required to reduce alcohol consumption to reduce longer-term health risks were considered “*too long to go for”* and therefore dismissed as unachievable. As the following participant explained, the risk message is subsequently ignored:

“*I don't think about the risk [of breast cancer] I put it to the back of my mind and carry on regardless, I still don't put enough emphasis on that connection and its bad, I know it's bad but I can't explain why because I figure that alcohol, the risk of alcohol can be damaging any part of you, not just the breast cancer connection you know. So, if I worry about breast cancer, I've got to worry about…. it's like If I don't think about it then it's not going to hurt me so I won't stress about it and if it happens it happens…so it's almost like worry about what's happening right now and worry about the things you can actually control if you know the dangers” (Danielle, aged 46, light drinker, separated, children, during COVID-19)*.

Earlier we explained how participant Anna (a self-perceived moderate drinker before COVID-19) focused her risk perceptions on the short-term virus prevention, not alcohol-related health outcomes. She later clarified that her intentions to reduce consumption during COVID-19 and focus on health resulted in moments of overwhelm and ultimately denial:

“*So, probably that – when you go, well, or you're a bit anxious about what's going on, and then, you know, put your health into focus, then the flow-on effect of that is that every now and then is, “I've had enough, I just want to – I just want to relax and remember what it's like when I don't have to worry about these things. So, that's when alcohol is probably brought in” (Anna, aged 46, moderate drinker, married, children, during COVID-19)*.

Several participants' curiosity about reducing their alcohol consumption before COVID-19, found that motivation was quashed during the pandemic. This is captured through our interview with Danielle who was preparing to reduce her alcohol consumption by researching her risk levels before COVID-19:

“*I actually did a web search to see what I could do if I wanted to reduce the amount of drinking and then I come across this website in the UK and it actually got you to put in just some basic details, you know, age group and sex and all that sort of thing, to see if they consider your drinking unsafe and I was” (Danielle, aged 46, light drinker, separated, children, before COVID-19)*.

However, during the pandemic, she explained that any plans to reduce consumption were on hold due to anxiety associated with COVID-19:

“*I have done (thought about the risks of drinking) but certainly not while this is going on [COVID-19] while this is going on I'm just going to continue to behave as normally as possible…so I'm not in that anxious state I'm just going to keep doing…I've been trying to behave as normally as possible to beat the potential [COVID-19] anxiety” (Danielle, aged 46, light drinker, separated, children, during COVID-19)*.

## Discussion: Relevance and Implications for Risk Messaging

The COVID-19 pandemic has highlighted that Australian women's alcohol consumption and perceptions of health risk are shaped by social practises across a range of life circumstances – economic, social, cultural, and emotional. Many participants in our study, who consumed alcohol prior to COVID-19, rationalised their consumption in new ways in terms of risk and according to the demands of the immediate situation during COVID-19. In other words, context, which included the presence of COVID-19 in the community, and the associated lockdown and social distancing requirements, defined the short-term context in which the decision to consume was made. COVID-19 created great uncertainty for many Australians. As uncertainty is exacerbated, achieving a sense of personal control might increasingly involve focus on coping with difficulties in the here and now. Consequently, alcohol consumption might be influenced by pragmatic considerations including the management of acute needs. Our results suggest that times of crisis, like the COVID-19 pandemic, orientates women toward the demand of the immediate future. This involves assessing and responding to more urgent, daily probabilities, where risk perceptions are refocused on the “short horizon” (18). The women we interviewed during COVID-19 described having developed a reduced “headspace” for consideration of long-term health risks, including acting on the association between alcohol consumption and breast cancer risk (as an example of a longer-term health risk linked to alcohol consumption). Here we are reminded of Beck's “eschatological ecofatalism” (32) to describe how the constant uncertainty experienced through COVID-19 and feeling of no certainty, and of avoiding “danger,” might result in women “pulling the shutters down” to risk and avoid contemplating risk altogether (33). We are also reminded of the dichotomous, “good/bad” tensions within coping strategies that allow immediacy in allowing relief during adversity – a sense of “doing what it takes” to get through the difficult short-term albeit compromising longer-term health outcomes (34).

The inverse relationship between perceived benefit and perceived risk of an action whereby people judge a risk not only by what they think about it but how they feel about it (35) has relevance for how we might understand women's weighing up of the costs and benefits of alcohol consumption during COVID-19. It is clear in our interview data collected during COVID-19 compared to women's responses before the pandemic, that the feelings women experience amidst COVID-19 provide “information” on which judgements about risk should/do inform decision-making. It is also likely difficult for women to see this as problematic given the extent to which alcohol is embedded in the social fabric of Australian society and considering norms that tell women consuming it is a normal, socially acceptable coping mechanism. This is crucial for the delivery of health risk messaging during COVID-19, and any times of crisis – it is futile to tell women about the longer-term health risks of alcohol while they are preoccupied with simply “getting through” and the associated immediate needs. Leverage in alcohol-related health promotion will have more potential if there is acknowledgement of how consumption behaviours are shaped by, and integrated within, women's daily living, which is naturally tied to their ongoing and dynamic perceptions of risk. It must be acknowledged that monitoring personal alcohol consumption is challenging whilst surrounded by alcohol product marketing that targets women (36), living in an environment where alcohol remains readily available during COVID-19 lockdown (37) (e.g., liquor stores remained open albeit with some restrictions including volume limits on purchases and limited gatherings in licenced premises), and where consuming alcohol is marketed as a socially acceptable way to manage day-to-day stress during the pandemic^2^ and to celebrate the lifting of restrictions (38). As we have noted elsewhere (8), women's appraisal of their own alcohol-related health risk culminates from information brought together through personal, social, and cultural sources.

Our findings also demonstrate that different women have been impacted differently by COVID-19, in terms of their alcohol-related risk perceptions, and seem to be linked to their life circumstances before but also during COVID-19 (according to the impact of the pandemic). The “collective struggle” to adapt and modify our lives has been necessary to “flatten the epidemiological curve.” However, population level distancing measures to lower mean risk levels have entailed different modifications for different groups of women. Existing social and economic vulnerabilities mean personal costs and private misfortunes are amplified through pandemic countermeasures (39, 40), in turn affecting possibilities to reduce alcohol consumption. This has been made visible through our discussions with women that show uncertainty with the possibility of inflating or solidifying risk perceptions was experienced by some women during COVID-19. Yet, our research also clarifies that for some women life during the pandemic resembles their already chaotic and unpredictable lives. It is likely that certain women have been, and will continue to be, disproportionately impacted economically, emotionally, socially by government requirements to socially distance during COVID-19, in turn impacting their alcohol-related decision-making differently relative to men (39). Of course, it must be acknowledged that our study recruited women of mainly Anglo-Saxon ethnicity and there are some limitations to the generalizability of our understandings reported here to the broader population of Australian women. We intend to undertake additional analyses that would differentiate women's responses by social class groups and achieve a more complete understanding of women's alcohol consumption and risk perceptions during COVID-19 according to demographics. This would allow insight to whether changes in perceptions of the risk of alcohol are impacted differently during COVID-19 depending on women's social class grouping. This would also enable theoretically-informed explanations of class-based experiences and contribute to understanding population-level distributions of risk factors and perceptions amongst groups of women (41, 42). How this might translate to class-based alcohol-related risk communication is uncertain, though relevant and is worthwhile given we do not know the lasting effects of this pandemic and when the future is uncertain, future-oriented public health interventions that rely on individual action are limited. Beyond the immediate risk of contracting the virus, it is not clear what the prolonged social and economic impacts will be on well-being and the ongoing bearing on women's future alcohol consumption and overall perceptions of health risks. As Australia shifts focus toward the recovery phase of the COVID-19 pandemic and the future remains unclear, our findings have relevance for future public health approaches that respond to COVID-19. As countermeasures shift and change in response to the pandemic, women's responses, and ways of coping manifest through alcohol consumption, might change over time. Gendered policy responses are needed that consider the implications for risk messaging as experienced specifically by women, as a population group vulnerable to the unintended consequences of pandemic social distancing measures described herein.

## Conclusion

This paper outlines to policy makers the impact of current and future COVID-19 interventions on women's alcohol consumption as well as their shifting perceptions of health risks. It is useful for planning future policy to mitigate the unintended consequence of pandemic responses in terms of links to increased alcohol consumption. For the South Australian women we interviewed, pandemic responses required focusing on surviving the “crisis” in the immediate future, a sense of doing whatever is required to get by, and the short-term gains of alcohol consumption in this context overshadow any longer-term health risks. By understanding this logic, we can identify strategies to change women's present alcohol consumption to potentially improve their health outcomes into the future. We can also recognise how individual health risks are moored within and contextualised by broader social structures that warrant policy consideration.

## Data Availability Statement

The raw data supporting the conclusions of this article will be made available by the authors, without undue reservation.

## Ethics Statement

Written informed consent was obtained from the individual(s) for the publication of any potentially identifiable images or data included in this article.

## Author Contributions

BL collected the data and undertook the data analysis. KF collected the data and reviewed the data analysis. JT, MW, and PW reviewed the data analysis. BL wrote the first draft of the manuscript and all authors participated in the development and editing of the manuscript, including the final approval. All authors contributed to the design of the study and its analytical approach.

## Conflict of Interest

The authors declare that the research was conducted in the absence of any commercial or financial relationships that could be construed as a potential conflict of interest.

## Footnotes

^1^See: https://www.covid-19.sa.gov.au/response.

^2^For examples see: https://www.abc.net.au/news/2020-04-16/coronavirus-themed-alcohol-marketing-sparks-call-for-ban/1215191.
